# Assessment of Hepatic Profile in Acquired Aplastic Anemia: An Experience From Pakistan

**DOI:** 10.7759/cureus.29079

**Published:** 2022-09-12

**Authors:** Warkha Thakur, Nida Anwar, Shafaq Samad, Naveena Fatima, Rehana Ahmed, Faryal Tariq, Javeria Ashfaq, Sumaira Sharif, Munira Borhany

**Affiliations:** 1 Hematology, National Institute of Blood Diseases & Bone Marrow Transplantation, Karachi, PAK; 2 Hematology and Bone Marrow Transplantation, National Institute of Blood Diseases & Bone Marrow Transplantation, Karachi, PAK; 3 Bone Marrow Transplantation, National Institute of Blood Diseases & Bone Marrow Transplantation, Karachi, PAK; 4 Research and Development, National Institute of Blood Diseases & Bone Marrow Transplantation, Karachi, PAK; 5 Clinical Hematology, National Institute of Blood Diseases & Bone Marrow Transplantation, Karachi, PAK; 6 Clinical Hematology, Jinnah Postgraduate Medical Centre, Karachi, PAK

**Keywords:** acquired aplastic anemia, pakistan, hepatitis associated aplastic anemia, hepatitis, aplastic anemia

## Abstract

Introduction: Aplastic anemia (AA) is characterized by pancytopenia and hypocellular marrow in the absence of an abnormal infiltrate or increase in reticulin fibrosis. The diagnosis of AA is challenging at times due to decreased cellularity and overlapping morphological features with other bone marrow failure syndromes. Hepatitis-associated aplastic anemia (HAAA) is a rare variant in which patients typically present with jaundice and hepatitis followed by pancytopenia almost within 6 months. Post-hepatitis AA accounts for approximately 1-5% of cases, and invariably such cases are negative for the known hepatitis virus as well. There is limited literature available to understand the correlation of AA with hepatitis with none reported at the national level in our region. As AA is relatively more prevalent in Southeast Asia as compared to the western world and hepatitis is a prevalent disease in our population, the main purpose of this study was to assess the hepatic profile and determine the association of hepatitis in AA at the time of diagnosis.

Materials and methods: A cross-sectional study was carried out at the National Institute of Blood Disease and Bone Marrow Transplantation, Karachi, from November 2019 to December 2020 after the informed consent from patients. The study included all treatment-naïve patients of acquired AA with no prior history of taking steroids, immunosuppressive treatment, or chemoradiation therapy. Liver function tests, complete blood count, prothrombin time (PT), and activated partial thromboplastin time were performed, along with viral profiles (HAV, Hep B, Hep C, and HIV). SPSS version 23 (IBM Corp., Armonk, NY) was used for statistical analysis. Mean and standard deviations were computed for quantitative variables while percentages and frequencies were reported for qualitative variables. T-test was used to observe the main difference between groups and a p-value <0.05 was considered to be significant.

Results: Out of a total of 351 patients, 29 (8.2%) patients with AA tested positive for viral hepatitis. Hepatitis A was the most prevalent hepatitis (4.0%), followed by hepatitis C (3.7%). The comparison of platelet counts in patients with and without hepatitis was reported to be of statistical significance (p-value < 0.05). A significant statistical difference (p-value < 0.0001) was found in platelet count and PT in patients of AA with and without hepatitis.

Conclusion: Overall, this study revealed that <10% of patients of AA had a positive screening for hepatitis A, B, and C and low platelet count, and PT was statistically significant when compared between the patients with and without hepatitis. Hepatitis being prevalent in our part of the world might have an important causal association with AA. Patients with AA should be screened for liver functions and viral hepatitis at the time of diagnosis. In addition to hepatitis A, B, and C and HIV, other causes of hepatitis should also be screened such as parvovirus B19, human herpes virus 16, and adenovirus which are not included in routine diagnostic viral testing panel.

## Introduction

The distinctive manifestation(s) of aplastic anemia (AA) are pancytopenia and hypocellular bone marrow without evidence of infiltration, dysplasia, and fibrosis. It is caused by several risk factors including infections, toxins, chemotherapeutic drugs, and radiation but the precise cause remains unclear [1]. An uncommon form of AA known as hepatitis-associated aplastic anemia (HAAA) occurs when pancytopenia develops simultaneously with or within six months after an elevated serum alanine aminotransferase (ALT) level. Significantly, these ALT levels are five times higher than the upper limit of normal. Post-hepatitic AA accounts for approximately 1-5% of cases invariably, and such cases are negative for the known hepatitis virus as well [2]. When compared to patients with non-hepatitis-associated AA in the pediatric population, those with HAAA have considerably lower survival and prognosis [3]. Hepatitis symptoms linked to HAAA can be self-limiting, but sometimes show moderate to severe or acute to the chronic clinical course [4]. Necrosis of the portal region and fibrosis that extends up to the centrilobular area can be found in the transjugular liver biopsy of HAAA patients and chronic severe hepatic inflammation quickly progresses to liver fibrosis [5]. First-line therapies include stem cell transplantation from a sibling donor who matches the patient's human leukocyte antigen (HLA) profile or immunosuppressive therapies like cyclosporine and/or antithymocyte or antilymphocyte globulin [6,7].

The exact etiology of hepatitis causing AA is nearly unknown and the pathogenesis of HAAA has been associated with activated T1 cells [8]. Hepatitis B and C virus, parvovirus B19, human herpes virus 16, and transfusion-transmitted virus (hepatitis B, C, HIV) can all be causal associations. The traits include CD8+-predominant lobular necroinflammatory and endothelial damage associated with sinusoidal obstruction syndrome, conjugated hyperbilirubinemia, elevated antinuclear antibody titers, and elevated transaminases [9]. Dietary or nutrition supplements at times may also result in toxin-induced hepatitis [10]. It is typical for intrabiliary cholestasis and liver toxicity to result in the apoptotic killing of hematopoietic cells by CD8 lymphocytes and T cell-induced gamma interferon, which leads to hepatitis. Clinical symptoms include pallor, exhaustion, petechial rash, and infections due to pancytopenia [11]. When severe thrombocytopenia and anemia appear at the same time as HAAA, it is important to distinguish this condition from infantile giant cell hepatitis with autoimmune hemolytic anemia [12].

Allogeneic bone marrow transplantation (allo BMT) is the conventional curative treatment for HAAA from an HLA-matched donor. Since HAAA has a poor prognosis, allo BMT has been the curative form of treatment [13-15]. On the other hand, immunosuppressive medication results in a response rate of 70% and a survival rate of 85% for patients not receiving hematopoietic cell transplantation, respectively [16]. Children respond to BMT better than adults do, and the survival rates of patients receiving bone marrow from HLA-matched donors are found to be comparable to that of the patients with AA not caused by hepatitis [17]. There is no recognized antiviral medication for hepatitis B-related HAAA. Lamivudine, a nucleoside analog, has been studied for use in treating hepatitis B-related HAAA, and it has been shown to be effective in causing remission in cases of severe AA and hepatitis B virus infection. Because of its myelosuppressive effects, interferon, an acclaimed antiviral agent in the treatment arsenal of HBV- and HCV-mediated infections, cannot be used as a treatment option for the HAAA [18]. Patients with HAAA exhibit severe pancytopenia following an episode of acute hepatitis, and if left untreated, the marrow suppression is frequently swift and severe.

In Pakistan, cases of hepatitis have been increasing over time, and given the current policies and practices, eradicating hepatitis from Pakistan by 2030 seems unreasonable [19]. We have also noted a rising trend in the incidence of AA than reported internationally. The association of hepatitis with AA is underreported and limited literature is available regarding the determinants of AA. Hepatitis might be one of the causes of AA and it must be considered an alarming situation. The only curative option for AA is BMT, which is an unaffordable treatment option for many patients in Pakistan. Therefore, the aim to conduct this study was to evaluate the hepatic status in relation to hepatitis in treatment-naïve AA patients.

## Materials and methods

This was a cross-sectional study carried out at the National Institute of Blood Diseases and Bone Marrow Transplantation (NIBD & BMT). Patients were recruited between November 2019 and December 2021. Prior to the initiation of the study, approval was taken from the NIBD ethics committee bearing NIBD/RD-188/11-2019 and informed consent was taken from the participants prior to enrollment in the study. The study included all treatment-naïve patients of acquired AA with no prior history of taking steroids, immunosuppressive treatment, or chemoradiation therapy. Patients with positive chromosomal breaks (Fanconi’s anemia) or inherited bone marrow failure were excluded. WHO sample size calculator was used to calculate the sample size, and by taking the percentage reported in the literature (5%) [14] it was found to be at least 73, and hence we recruited 351 patients who visited during the study duration. Non-probability purposive sampling method was used for patient selection. Demographic and laboratory parameters were recorded through a structured questionnaire and the diagnosis of AA was confirmed on bone marrow biopsy. Liver function tests (LFTs), complete blood count (CBC), prothrombin time (PT), and activated partial thromboplastin time (APTT) were performed. PT and APTT were run on STAGO and Sysmex CA-1500, and CBC was performed using a Sysmex XN-1000 analyzer from the Sysmex Corporation in Kobe, Japan. LFTs were performed on a Cobas C 111 analyzer machine that runs on a spectrophotometer principle. Within 2 hours of the blood sample collection, an aseptic setting was used to take a 4 ml blood sample from each patient, which was then tested for the presence of hepatitis A, HBV, HCV, and HIV. Detection of hepatitis B surface antigen and anti-hepatitis C antibodies was done using the chemiluminescence technique. To confirm chronic HCV and HBV infection in individuals with positive antibody tests, a qualitative nucleic acid test was employed as the initial diagnosis of a suspected acute infection. Ultrasound was done to complement the diagnosis of hepatitis. The data were entered in MS Excel and analysis was done by using Statistical Package for Social Sciences (SPSS) version 23.0 (IBM Corp., Armonk, NY). Shapiro-Wilk test was performed for normality and data were found normally distributed. Mean and standard deviations were computed for quantitative variables and percentages and frequencies were reported for qualitative variables. T-test was used to observe the mean difference between the two groups and a p-value <0.05 was found to be significant statistically.

## Results

A total of 351 patients were enrolled, out of which 222 (63%) were males, 265 (76%) were unmarried, 11 (3.1%) had hypertension, and 9 (2.5%) had diabetes. The mean age of participants was 30.9 ± 27.5. Twenty-nine (8.2%) patients with AA had hepatitis. The most common hepatitis was hepatitis A, 14 (4.0%), followed by hepatitis C, 13 (3.7%). The most common form of hepatitis C was acute 9 (69.2%). Two patients had compensated cirrhosis and two patients had decompensated cirrhosis (Table 1).

**Table 1:** Hepatitis status of participants

Hepatitis status of patients	N (%)
No history of hepatitis	322 (91.4%)
Hepatitis A positive	14 (4.0%)
Hepatitis B positive	2 (0.6%)
Hepatitis C positive	13 (3.7%)
Acute hepatitis	9 (69.2%)
Chronic compensated hepatitis	2 (1.5%)
Chronic decompensated hepatitis	2 (1.5%)

The association of CBC parameters such as hemoglobin, red blood cell count, packed cell volume, mean corpuscular volume, mean corpuscular hemoglobin, mean corpuscular hemoglobin concentration, total leukocyte count, neutrophils, lymphocytes, monocytes, and platelets with the patients with and without hepatitis was assessed and it was found out that platelet counts were statistically significant (p-value: 0.0020) as shown in Table 2.

**Table 2:** Comparison of CBC profile in participants with and without hepatitis CBC: complete blood count; Hb: hemoglobin; RBC: red blood cell count; PCV: packed cell volume; MCV: mean corpuscular volume; MCH: mean corpuscular hemoglobin; MCHC: mean corpuscular hemoglobin concentration; TLC: total leukocyte count

CBC parameters	With hepatitis (mean ± SD) (n = 22)	Without hepatitis (mean ± SD) (n = 322)	p-Value
Hb (g/dL)	7.4 ± 2.0	8.1 ± 4.6	0.520
RBC (×10^12^/L)	2.4 ± 0.9	4.7 ± 20.3	0.60
PCV (%)	27.5 ± 13.9	24.2 ± 11.1	0.195
MCV (fL)	81.9 ± 21.6	86.3 ± 15.9	0.227
MCH (pg)	29.9 ± 2.8	31.9 ± 23.7	0.687
MCHC (g/dL)	32.2 ± 6.4	32.7 ± 6.6	0.730
TLC (×10^9^/L)	2.9 ± 1.1	2.9 ± 1.6	0.954
Neutrophils (%)	18.7 ± 14.5	20.4 ± 19.5	0.695
Lymphocytes (%)	63.3 ± 26.8	52.8 ± 33.7	0.155
Monocytes (%)	3.7 ± 3.6	3.1 ± 3.2	0.386
Platelets (×10^9^/L)	29 ± 28	4 ± 3	0.002

Table 3 depicts the association of liver profile such as serum glutamate pyruvate transaminase, gamma glutamyl transferase (GGT), serum glutamic oxaloacetic acid transaminase, PT, APTT, and international normalized ratio with the patients with and without hepatitis, and it was found out that PT (p-value < 0.0001) was statistically significant.

**Table 3:** Comparison of liver profile in participants with and without hepatitis SGPT: serum glutamate pyruvate transaminase; GGT: gamma glutamyl transferase; SGOT: serum glutamic oxaloacetic acid transaminase; PT: prothrombin time; aPTT: activated partial thromboplastin time; INR: international normalized ratio

Liver profile	With hepatitis (mean ± SD)	Without hepatitis (mean ± SD)	p-Value
SGPT (U/L)	62.7 ± 50.1	78.8 ± 45.7	0.605
Alkaline phosphatase (IU/L)	262.5 ± 169.1	214.6 ± 142.7	0.133
GGT (IU/L)	22.0 ± 11.3	26.1 ± 15.2	0.071
SGOT (U/L)	62.5 ± 89.2	60.1 ± 85.1	0.896
Albumin (g/dL)	3.4 ± 2.1	3.6 ± 2.4	0.671
PT (test) (s)	17.0 ± 5.2	14.1 ± 2.7	<0.0001
aPTT (test) (s)	30.0 ± 10.2	30.7 ± 4.6	0.489
INR	1.5 ± 0.9	1.6 ± 1.1	0.549

## Discussion

In our study, 8.2% of AA patients had hepatitis. This is comparatively high as compared to the prevalence of hepatitis in the general population in Pakistan, which is reported to be around 4% [20]. A retrospective study carried out in Europe from 1997 to 2007 demonstrated that HAAA patients had a slight male predominance with a value of 68% [21], which is close to the prevalence of male participants in our study, i.e. 63.2%. This European study also demonstrated that there was no causative virus for hepatitis in 94% of HAAA patients. However, 15 patients (6%) had hepatitis with nine having hepatitis B virus and six having hepatitis A virus [21]. Similar results have been observed in our study with 91.4% HAAA serologically negative and not having any signs or symptoms of hepatitis. Twenty-nine patients (8.3%) had evidence of hepatitis: hepatitis A virus in 14 patients, hepatitis B virus in 2, and hepatitis C virus in 13 patients. Assessing the association of hepatitis C virus with HAAA, a study conducted in France showed that 15.8% of HAAA patients had hepatitis C virus [22], which was approximately three-fold higher compared to our study suggesting a prevalence of 3.7% of HAAA patients with hepatitis C virus. On the contrary, slightly similar figures were recreated in another comparative study done in Thailand which showed that 5.7% of the patients who were never transfused were found to have hepatitis C virus [23].

During the initial course of hepatitis, cytotoxic T lymphocytes (CTLs) occupy the same receptor antigen between liver and bone marrow cells. These CTLs replicate and extend to destroy bone marrow hematopoietic stem cells and result in AA [24,25]. However, another interesting finding of our study was that AA patients with hepatitis had considerably high platelet counts compared to those without hepatitis (29 ± 28 vs 4 ± 3). This finding is also in concordance with a study conducted by Wang WH et al. [3] in which the HAAA group had a considerably higher platelet count compared to the non-hepatitis-associated AA (50 × 10^9^/L vs 12 × 10^9^/L).

Literature reveals that among the most frequently assessed values in HAAA, there is a significant rise in ALT, GGT, and serum alkaline phosphatase [26]. The elevated levels are also shown in our results (Table 3). Considerable discrepancies were observed in PT in AA patients with and without hepatitis. This could be on account of viral hepatitis causing deranged LFTs [27]. However, studies comparing the levels of liver enzymes between hepatitis-associated and non-hepatitis-associated AA are not extensively reported in the literature.

Limitations

To the best of our knowledge, this is the first study at the national level to determine the association of hepatitis in patients with AA. However, the limitations of our study were that it was of cross-sectional nature and included non-probability sampling. Prospective cohort studies with stringent inclusion and exclusion criteria, a large sample size, and a controlled environment are needed to validate the findings.

## Conclusions

AA is a heterogeneous disease with a limited approach to curative treatment options like allogeneic stem cell transplant in our region. Hepatitis being prevalent in our part of the world might have an important causal association with AA. Patients should be screened for viral hepatitis at the time of diagnosis. Moreover, other causes of hepatitis should also be screened at the time of diagnosis such as parvovirus B19, human herpes virus 16, and adenovirus. Based on the rapidly advancing research methodologies, it is necessary to comprehensively analyze the underlying mechanisms of HAAA.
